# The Plague in Russia

**Published:** 1879-02-20

**Authors:** 


### *THE PLAGUE IN RUSSIA.*

A disease of appalling fatality has broken out in Eastern Europe, which has spread alarm and consternation on the Volga river and in Southeastern Russia. Rigid quarantine has been established against Astrakan, where it originated, but the panic-stricken inhabitants of that city are fleeing and scattering the infection in all directions.

